# Prevalence, Risk Factors, and Prognosis for Fontan-Associated Liver Disease: A Systematic Review and Exploratory Meta-Analysis

**DOI:** 10.1016/j.jacadv.2025.101694

**Published:** 2025-04-25

**Authors:** Jacob Y. Cao, Kathyrn M. Wales, Yves d’Udekem, David S. Celermajer, Rachael Cordina, Avik Majumdar

**Affiliations:** a Department of Cardiology, St. Vincent’s Hospital, Sydney, Australia; b Sydney Medical School, University of Sydney, Sydney, Australia; c Department of Cardiology, Liverpool Hospital, Sydney, Australia; d Division of Cardiac Surgery, Children’s National Hospital, Washington, District of Columbia, USA; e The George Washington University School of Medicine and Health Sciences, Washington, District of Columbia, USA; f Department of Cardiology, Royal Prince Alfred Hospital, Sydney, Australia; g Victorian Liver Transplant Unit, Austin Health, Melbourne, Australia; h Melbourne Medical School, University of Melbourne, Melbourne, Australia

**Keywords:** cirrhosis, hepatocellular carcinoma, single ventricle

## Abstract

**Background:**

Patients with Fontan circulation are at risk of progressive liver disease, but the prevalence and risk factors for Fontan-associated liver disease (FALD) remain unclear.

**Objectives:**

The aim of the study was to review unbiased data on FALD prevalence, diagnostic methods, risk factors, and prognostic significance, and to undertake exploratory meta-analysis on available data.

**Methods:**

This systematic review included studies with unselected FALD screening. Outcomes were imaging or biopsy-proven cirrhosis, advanced fibrosis, portal hypertension, and hepatocellular carcinoma. Exploratory meta-analysis was performed, as well as subgroup analyses and meta-regression to explore contributors towards outcome heterogeneity.

**Results:**

Thirty-seven studies comprising 5,701 patients were included, with a median of 17 years of follow-up post-Fontan completion. All estimates of FALD were highly heterogeneous, reflecting variable patient factors and institutional practices. Cirrhosis was diagnosed in 21% of patients, but ranged from 0% to 76%. Advanced fibrosis without cirrhosis was noted in 30%, portal hypertension in 17%, and hepatocellular carcinoma in 2%, also with significant heterogeneity. Subgroup analysis and meta-regression highlighted several factors that contributed to such heterogeneity. It was found that cirrhosis was less commonly diagnosed by biopsy than by imaging (10% vs 26%). Other risk factors for cirrhosis included years post-Fontan completion, atriopulmonary Fontan, moderate or greater ventricular dysfunction, and higher pulmonary capillary wedge pressure. Qualitative synthesis noted FALD to be associated with elevated risk of cardiovascular and all-cause mortality.

**Conclusions:**

Liver disease is common post-Fontan completion, though prevalence varies widely. Several risk factors should guide patient screening. A universal, prognostically meaningful FALD definition is needed to advance research and clinical care.

The Fontan circulation, also known as a univentricular circulation, is the result of several staged operations that redirect systemic venous blood flow into the pulmonary arteries without transiting through a functioning subpulmonic ventricle. While this circulation has significantly improved survival in patients with complex congenital heart disease^1^, cardiac and extra-cardiac complications continue to accrue over the lifetime of these patients due to their unique physiology. By necessity, systemic venous pressure is persistently elevated in this situation, and cardiac output is often low. These factors may have implications for the liver. Fontan-associated liver disease (FALD) describes a wide spectrum of progressive liver injury that is seen in these individuals.^2^ Despite being first described in the 1980s and subsequent ubiquitous use of the term in published literature, there remains no unifying definition.^3^^,^^4^ This likely stems from a combination of poor understanding of pathophysiology, biased prevalence data, unvalidated diagnostic methods, and unclear prognostic significance of FALD. Similar issues were highlighted in a 2017 expert statement, describing these as major barriers toward effective screening and treatment strategies.^2^

Point prevalence estimates of FALD vary widely in the literature, in part due to selection bias of the cohort being studied.^5^^,^^6^ Since liver follow-up protocols differ between centers, so too does the pretest probability of patients screened for FALD. A range of diagnostic methods with different thresholds for FALD has also been described, which further exacerbates the highly variable prevalence estimates. Diagnostic tests have included serological panels, imaging parameters, or histopathology, and while validated in the other causes of cirrhosis, their value in FALD is unclear. To date, there has been no comprehensive review of the existing FALD literature to qualitatively and quantitatively summarize what is known, and in doing so, to highlight the current issues that should be addressed in forthcoming research. Therefore, the aim of the current systematic review is to present an unbiased overview of the prevalence, diagnostic methods, risk factors, and prognostic significance of FALD. An exploratory meta-analysis was also performed to evaluate factors that underpin the highly heterogenous prevalence estimates in the literature and that may be useful in individual patient risk stratification.

## Methods

### Literature search strategy

A systematic review and meta-analysis was performed. The Preferred Reporting Items for Systematic Reviews and Meta-Analyses guidelines were followed.^7^ Electronic searches using Medline, PubMed, EMBASE, Cochrane Central Register of Controlled Trials, and Cochrane Database of Systematic Reviews were undertaken to include all relevant studies up to September 2023. A search strategy was devised to maximize sensitivity (Supplemental Appendix), combining synonyms and variations of “Fontan OR total cavopulmonary anastomosis” AND “cirrhosis OR portal hypertension OR hepatic fibrosis OR hepatocellular carcinoma.” The search was limited to human subjects only. No limit was set on language. Additionally, reference lists of retrieved articles and reviews were examined for further studies. European, American, and Australian trial registries were also searched for relevant studies (www.anzctr.org.au; www.clinicaltrials.gov; www.clinicaltrialsregister.eu). Where studies were from the same center that reported on the same outcomes, the one with the largest cohort size was used. Where a multicenter study was included, any other study from one of the participating centers was excluded. No ethics committee approval was required for the current systematic review.

### Selection criteria and data extraction

To be included for statistical synthesis, individual studies had to meet the following criteria: 1) inclusion of patients with Fontan circulation; 2) report at least one clinical outcome of interest as described below. Studies were excluded if: 1) liver screening was selectively undertaken for those with pre-existing or suspected liver disease; 2) there were less than 5 patients; and 3) containing data from registries without specifying participating centers resulting in the risk of duplication bias. Where several studies arose from the same research group, only the one with the largest cohort was included. Duplicate studies from the same group were only included if they reported on mutually exclusive outcomes (eg, cirrhosis and portal hypertension).

Nonselective methods of liver screening included 1) consecutive recruitment; 2) screening as part of institutional protocol; and 3) screening patients based on criteria that are unlikely related to FALD risk (eg, geographic location). Examples of selective screening include evaluating only patients with suspected liver disease based on clinical or biochemical abnormalities, preheart or liver transplant patients, or patients with failing Fontan circuits. Where the selection protocol was not specified, the studies were included, but subsequent sensitivity analyses were done to evaluate their effect on the outcomes. Certain studies specified exclusion of other common causes of liver disease (eg, viral hepatitis and significant alcohol intake), but given that these are uncommon in a young Fontan population, studies that did not specify these exclusions were included for the current meta-analysis.

Title and abstract screening and data extraction were independently performed by 2 investigators (J.Y.C. and K.M.W.). Disagreements were resolved by a third author (R.C.). The primary outcome of interest was cirrhosis, and secondary outcomes were advanced fibrosis without cirrhosis, portal hypertension and hepatocellular carcinoma (HCC). There is significant variation in definition of each outcome (Supplemental Table 1). We excluded studies using only serological markers for diagnoses given different thresholds between studies as well as a lack of strong evidence for their use in FALD.^2^ Imaging and biopsy parameters used for diagnoses also varied between investigators but were reviewed by a senior hepatologist with experience in FALD (A.M.) for inclusion. Ultimately, all studies using imaging or biopsy were included given the lack of established diagnostic criteria and to maximize the representativeness of the systematic review. Additional baseline variables relating to study characteristics, patient demographics, cardiac function, and hemodynamics were extracted for post hoc subgroup analyses and meta-regression. Where actuarial incidence was not reported at a specific time point, data were extracted from available Kaplan-Meier curves using Plot Digitizer (https://plotdigitizer.com). For example, although Inuzuka et al^8^ reported 67 cases of cirrhosis at a median of 10.3 years of follow-up, it was unclear whether this was from Fontan completion or from the first contact with the study investigators pre-Fontan completion. As such, the presented Kaplan-Meier curves of post-Fontan cirrhosis prevalence were used instead to extract data at 15 years, at which point the study had a third of the original cohort still being followed up.

Exploratory statistical synthesis was not performed for FALD prognosis and management due to highly variable definitions and outcomes. Rather, available literature was qualitatively synthesized and presented. For FALD prognosis, all studies that evaluated association between any FALD parameters and clinical outcomes were selected. Clinical outcomes included all-cause mortality, cardiovascular mortality, heart or liver transplantation, ventricular assist device implantation, cardiovascular hospitalization, thromboembolic event, arrhythmias, protein-losing enteropathy, plastic bronchitis, and HCC. For FALD management, any study that reported any pharmacological or surgical strategies with associated outcomes was selected.

### Data analysis

Event rates were extracted for dichotomous outcomes and mean and SD for continuous outcomes. Where median and IQR or minimum-maximum range was reported, a validated method was used to convert to mean and SD.^9^ A random-effects model using a random-intercept logistic regression with maximum likelihood estimator was employed to account for differences in patient characteristics and procedural factors between studies.^10^ Subgroup analyses by diagnostic method and patient selection method and post hoc univariable meta-regression were performed to explore variables that might have contributed toward the heterogeneity in the summary effect sizes. Sensitivity analyses using the leave-one-out method were undertaken to assess the robustness of the outcomes.

Publication bias was assessed visually by funnel plots and statistically by Begg test. All statistical analyses were carried out using R Statistical Software (v4.3.1, R Core Team 2021).

## Results

The search results are shown in Supplemental Figure 1 in accordance with the Preferred Reporting Items for Systematic Reviews and Meta-Analyses flow chart. Thirty-seven studies were included for statistical synthesis, comprising 5,701 patients, followed up for a median of 17 years (IQR: 14-20 years) post-Fontan completion. Study characteristics are summarized in Table 1.

**Table 1:** Study and Patient Baseline Variables

First Author, Year	Selection	N	Diagnostic Method	Age at Fontan Completion, y	Fontan Type (%)	≥ Mod Ventricular Dysfunction (%)	≥ Mod AVVR (%)
Zentner et al, 2023^11^	Unselected	70	Imaging	5.3	AP 43, ECC 42, LT 15	12	
Zafar et al, 2022^33^	Unselected	123	Imaging	6.5	AP 15, ECC 44, LT 41	13	18
Inuzuka et al, 2022^8^	Unselected	1,117	Mixed	4			15
Gunsaulus et al, 2022^34^	Unclear	44	Imaging	3.03		8	6
Thrane et al, 2021^35^	Unselected	46	Biopsy^a^	2.6			
Shin et al, 2021^36^	Unclear	45	Biopsy	5.3	AP 27, ECC 24, LT 49		11
Sakamori et al, 2021^37^	Unselected	103	Mixed^a^	3	AP 11, ECC 66, LT 23		
Ohuchi et al, 2021^28^	Unselected	339	Biopsy^a^	2.3			
Navallas et al, 2021^38^	Unselected	37	Imaging	3.33	AP 0, ECC 97, LT 3		
Martinez-Quintana and Rodriguez-Gonzalez, 2021^39^	Unselected	14	Imaging	9.4			
Langguth et al, 2021^40^	Unselected	28	Imaging	2.9	AP 0, ECC 7, LT 93		
Hansen et al, 2021^41^	Unselected	240	Imaging	2.7	AP 1, ECC 11, LT 88		20
Emamaullee et al, 2021^42^	Unselected	106	Biopsy	3.5	AP 0, ECC 93, LT 7	7	29
De Bruyne et al, 2021^43^	Unselected	35	Imaging	3.34	AP 0, ECC 69, LT 31		6
Chemello et al, 2021^44^	Unselected	43	Imaging	3.5	AP 1, ECC 47, LT 44		
Anigwe et al, 2021^45^	Unclear	160	Imaging	7.07	AP 0, ECC 73, LT 27		28
Yoon et al, 2020^15^	Unselected	313	Imaging	2.9	AP 17, ECC 43, LT 40		
Wan et al, 2020^46^	Unselected	93	Imaging	7.9	AP 30, ECC 32, LT 38		18
Tellez et al, 2020^47^	Unselected	152	Biopsy^a^	9	AP 27, ECC 64, LT 9		
Sethasathien et al, 2020^48^	Unclear	80	Imaging	7.75	AP 3, ECC 98, LT 0		19
Abbasi et al, 2020^49^	Unclear	30	Imaging	11.9	AP 37, ECC 43, LT 20		3
SmasSuska et al, 2019^50^	Unselected	59	Imaging	7	AP 7, ECC 93, LT 0		19
Silva-Sepulveda et al, 2019^51^	Unselected	28	Imaging		AP 3, ECC 38, LT 59	2	4
Munsterman et al, 2019^52^	Unselected	38	Mixed	4.77	AP 39, ECC 42, LT 19	2	
Wilson et al, 2018^53^	Unselected	1,552	Biopsy^a^				
Song et al, 2018^5^	Unselected	26	Imaging	9.3	AP 0, ECC 96, LT 4	0	6
Schachter et al, 2018^54^	Unselected	14	Biopsy				
Nandwana et al, 2018^55^	Unclear	145	Imaging	4.25			
Kim et al, 2018^56^	Unclear	64	Imaging	5.4	AP 2, ECC 84, LT 14		
Egbe et al, 2018^57^	Unclear	164	Mixed	8			
Buendia et al, 2018^58^	Unselected	37	Imaging	6.3	AP 16, ECC 65, LT 19	7	6
Surrey et al, 2016^59^	Unselected	74	Biopsy	4.15	AP 5, ECC 34, LT 61		
Agnoletti et al, 2016^60^	Unselected	64	Mixed	8.83			11
Poterucha et al, 2015^23^	Unclear	50	Biopsy^a^	4	AP 40, ECC 26, LT 34		16
Lindsay et al, 2015^16^	Unclear	53	Imaging	6.65			
Wallihan et al, 2013^61^	Unclear	42	Imaging	9.53	AP 29, ECC 29, LT 43		
Elder et al, 2013^62^	Unclear	73	Imaging		AP 36, ECC 12, LT 52	12	Unclear

CVP (mm Hg)	PCWP (mm Hg)	PVR (WU)	MELD-XI	Follow-Up Post-Fontan (y)	Ref #
			29	24	11
13		1.43			33
12.8	7	2		15	8
13		1.43		16.2	34
				14.1	35
14				20.8	36
				19.6	63
10				25.6	28
					38
			24.3	16.5	39
				14.63	40
11					41
12.9		1.7	14.4	10.8	42
				7.31	43
					44
13.6	10.3	0.96		15.9	45
13			20	20	15
					46
14.9			10.8	18.3	47
15.3		3.3		8.43	48
17.25				18.85	49
			29	19.67	50
13	7.8	1.6		16.73	51
			27.6	21.4	52
				12.7	64
12.8		1.5		10.5	5
				24.7	54
				23.92	55
14.1		1.35		12.1	56
				26.67	57
			22.9	15.8	58
14				14.55	59
12	15.3	3	13.83	12.75	60
16	10	3	9	22	23
				18	16
				14.6	65
					62

AP = atriopulmonary; AVVR = atrioventricular valvular regurgitation; CVP = central venous pressure; ECC = extracardiac conduit; LT = lateral tunnel; MELD-XI = Model for End-stage Liver Disease excluding INR; PVR = pulmonary vascular resistance.

a Studies that reported hepatocellular carcinoma as the only liver-related outcome, so the diagnostic method refers to the diagnosis of the malignancy only.

Nineteen studies reported the primary pathology, comprising 1,715 patients. Tricuspid atresia was the most common in 571 (33%) patients, followed by hypoplastic left heart syndrome in 432 (25%), double inlet left ventricle in 203 (12%), pulmonary atresia in 135 (8%), and double outlet right ventricle in 108 (6%) patients. The median age at Fontan completion was 5.3 years (IQR: 3.3-7.8 years). Twenty-three studies reported on Fontan connection type, comprising 1,844 patients. This included extracardiac conduit in 981 (53%) patients, lateral tunnel in 661 (36%) patients, and atriopulmonary (AP) in 267 (15%) patients. Other baseline patient characteristics and presurgical and surgical variables are summarized in Table 1.

### Liver outcomes

**Figure 1:**
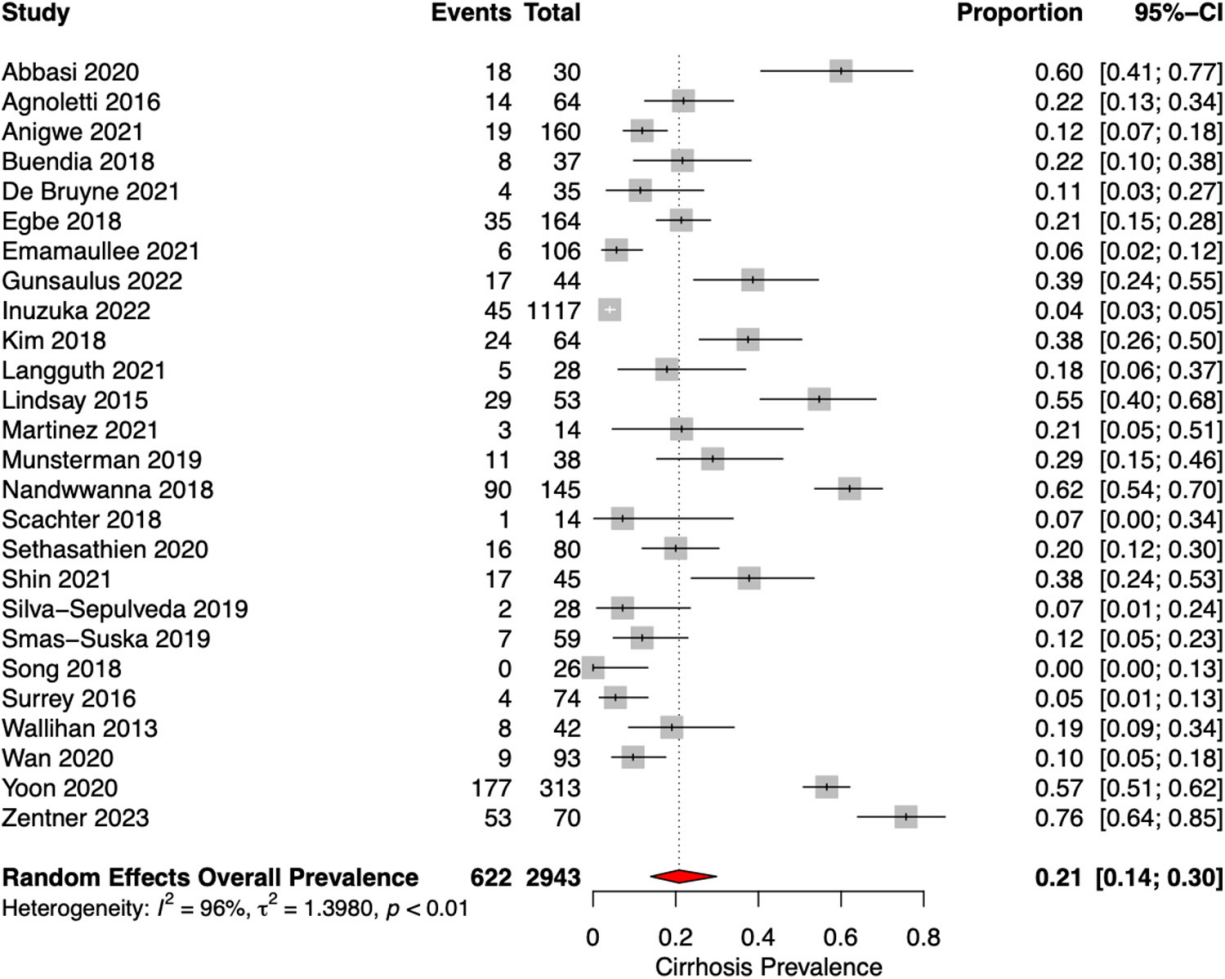
Prevalence of Cirrhosis Using Random-Effects Model

**Figure 2:**
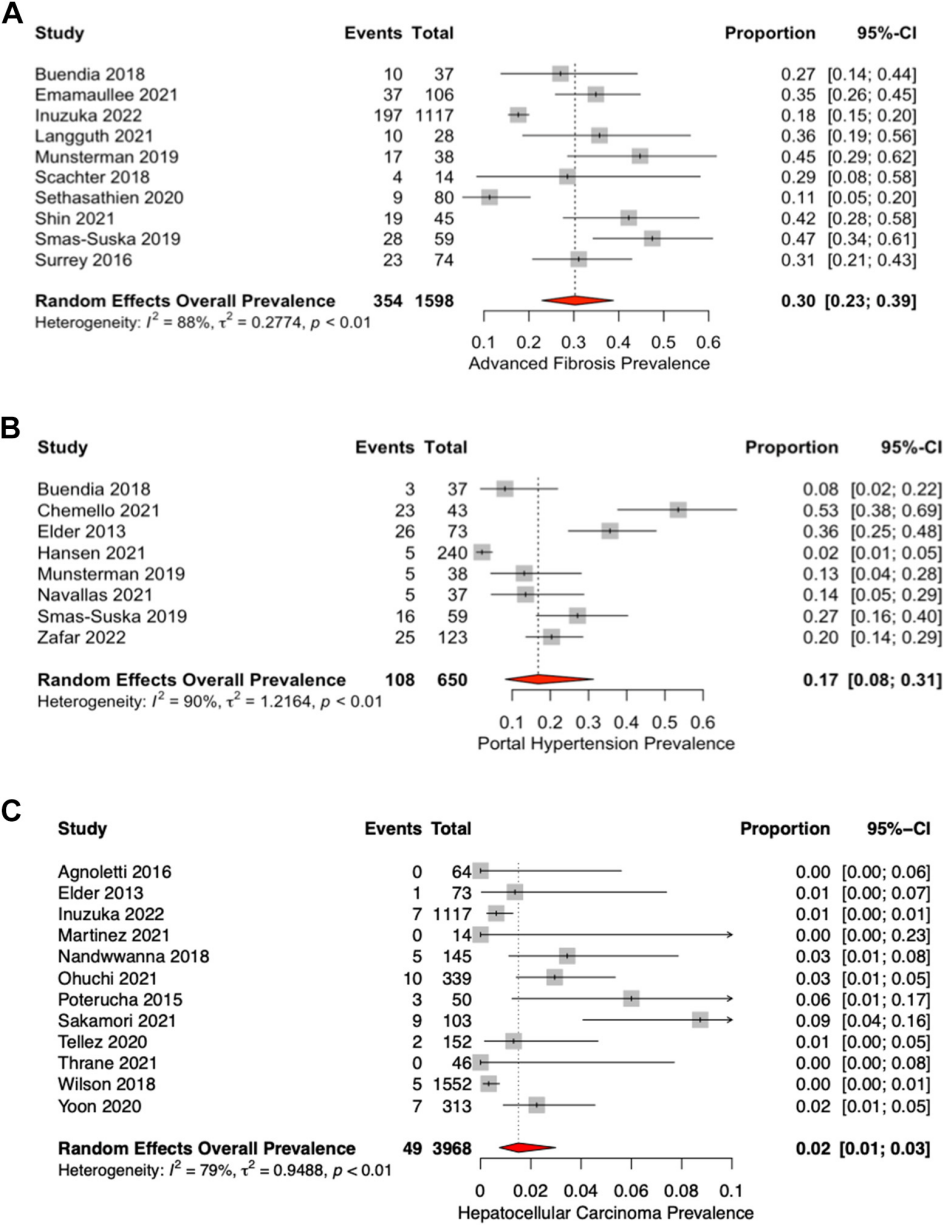
Prevalence of Advanced Fibrosis, Portal Hypertension and Hepatocellular Carcinoma (A) Prevalence of advanced fibrosis without cirrhosis, (B) portal hypertension and (C) hepatocellular carcinoma.

Cirrhosis, advanced fibrosis, and portal hypertension were defined in various methods between studies (Supplemental Table 1). HCC diagnosis was either undefined or by a combination of imaging or biopsy. Of the 26 studies reporting cirrhosis prevalence, there were 8 different, albeit overlapping, definitions of cirrhosis, and this was likewise observed for advanced fibrosis and portal hypertension. Overlapping imaging features included liver surface nodularity, heterogeneous echogenicity, and caudate lobe hypertrophy. Biopsies were scored using various systems that focused either on portal or centrilobular fibrosis. Cirrhosis was reported in 21% (95% CI: 13%-30%) (Figure 1) of the cohort at a median of 16 years follow-up post-Fontan completion, or approximately 47,000 patient-years. Advanced fibrosis without cirrhosis was reported in 30% (95% CI: 23%-39%; Figure 2A) of patients, portal hypertension in 17% (95% CI: 8%-31%; Figure 2B) and HCC in 2% (95% CI: 1%-3%; Figure 2C). The median follow-up post-Fontan completion for only studies reporting HCC was 18 years (IQR: 15-21 years).

Significant heterogeneity was noted in all 3 outcomes (*I*^2^ = 79%-96%, *P* < 0.01). The rate of cirrhosis ranged between 0% in Song et al^5^ with 10 years of follow-up to 76% in Zentner et al^11^ with 24 years of follow-up. Subgroup analysis by categorizing studies into those that have specified nonselective liver screening vs those without clear selection criteria noted a significantly lower rate of cirrhosis in the former group (14% vs 31%, *P* < 0.01) (Supplemental Figure 2). This implied that certain studies that have not specified selection criteria might have included a higher risk population for liver disease. Subgroup analysis by diagnostic method revealed that those studies with only biopsy-proven cirrhosis found a trend toward lower rate of cirrhosis than those with only imaging-proven cirrhosis (10% vs 26%, *P* = 0.09) (Supplemental Figure 3).

**Figure 3:**
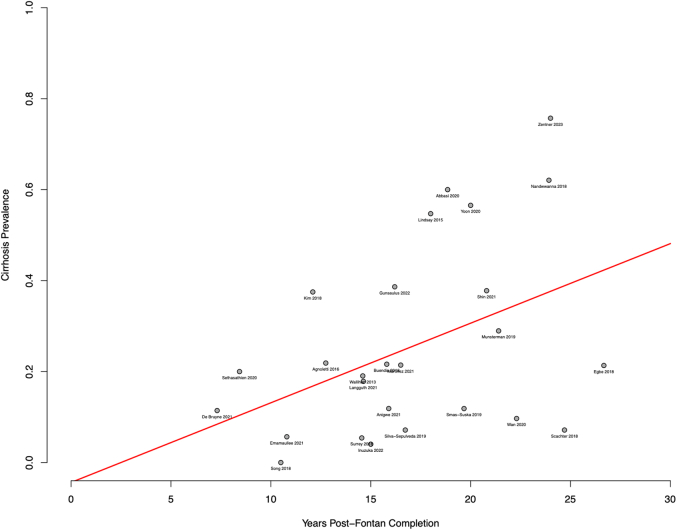
Positive Correlation Between Years Post-Fontan Completion and Cirrhosis Prevalence

Exploratory meta-regression evaluated potential predictors of cirrhosis. A linear relationship was found between years post-Fontan completion and odds of cirrhosis, although notably the shortest follow-up was 7.3 years, so whether a linear relationship exists before this is unclear. Every year post-Fontan completion conferred 11% increased odds of developing cirrhosis (95% CI: 1.02-1.22; *P* = 0.02; Figure 3) with the predicted prevalence of cirrhosis at 10, 15, and 20 years being 13%, 23%, and 33%, respectively. This relationship remained significant when including only studies specifying unselected liver screening in patients (OR: 1.02/year; 95% CI: 1.00-1.04; *P* = 0.04). Likewise, pulmonary capillary wedge pressure (PCWP), the proportion of AP Fontan, and of patients with at least moderate ventricular dysfunction were found to be predictors of cirrhosis (PCWP: OR = 1.27/mm Hg increase, 95% CI: 1.18-1.37; *P* < 0.001; AP Fontan: OR: 1.69/10% increase; 95% CI: 1.23-2.27, *P* < 0.001; ventricular dysfunction: OR: 5.9/10% increase; 95% CI: 1.2-29.6, *P* = 0.03) (Supplemental Figures 4 to 6). A trend toward a positive relationship was found between central venous pressure (CVP) and risk of cirrhosis (OR: 1.57/mm Hg increase; 95% CI: 0.95-2.61; *P* = 0.08) (Supplemental Figure 7). The Model for End-stage Liver Disease excluding INR score (MELD-XI) was not associated with the risk of cirrhosis in the current analysis (OR: 0.73; 95% CI: 0.41-1.27; *P* = 0.26). Results of univariable meta-regressions are summarized in Table 2.

**Table 2:** Univariable Meta-Regression Between Baseline Presurgical and Surgical Variables and Risk of Cirrhosis

	N	OR (95% CI)	*P* Value
Years post-Fontan completion (per year)	26	1.11 (1.02-1.22)	**0.02**
Gender (per 10% increase in male)	25	0.84 (0.58-1.23)	0.37
Age at Fontan completion (per year)	24	1.01 (0.81-1.22)	0.94
CVP (per mm Hg)	13	1.57 (0.95-2.61)	0.08
PVR (per Woods unit)	9	1.19 (0.45-3.12)	0.73
PCWP (per mm Hg)	4	1.27 (1.18-1.37)	**<0.001**
Fontan type (per 10% increase in AP proportion)	18	1.69 (1.23-2.27)	**<0.001**
Fenestration (per 10% increase)	13	1.04 (0.69-1.58)	0.85
MELD XI (per score)	8	0.73 (0.41-1.27)	0.26
≥Moderate ventricular dysfunction (per 10% increase in proportion)	7	5.91 (1.18-29.61)	**0.03**
≥Moderate AVVR (per 10% increase in proportion)	14	0.90 (0.42-1.93)	0.79

Bold values denote *P* < 0.05.

N = number of studies; PCWP = pulmonary capillary wedge pressure; other abbreviations as in Table 1.

### Prognosis and management strategies

Various studies have evaluated the prognostic value of liver injury on cardiovascular and hepatic outcomes, as well as overall mortality. However, due to the significant hetereogeneity in the prognostic markers and outcomes evaluated, statistical synthesis was not possible. As such, Table 3 systematically summarizes the existing data, with most studies suggesting that liver injury portends unfavorable long-term clinical outcomes including all-cause mortality, need for heart transplantation or ventricular assist device, and decompensated heart failure. In contrast, only 3 studies on management strategies of FALD were identified. Glenn et al^12^ examined the effect of phosphodiesterase type 5 inhibitors on histological progression of FALD and found no consistent effect, although this was severely limited by low number of patients with pre- and post-therapy biopsies. Two further case series of isolated heart transplant recipients found overall improvement in fibrosis features on imaging post-transplant.^13^^,^^14^

**Table 3:** Summary of Studies Evaluating Prognostic Role of Liver Disease in Fontan Patients

Diagnostic Method	Endpoint	Association, Effect Estimator/Size, *P* Value	Ref #
Imaging			
Ultrasound			
Liver volume/BSA	• Death, heart/liver transplantation	Higher risk ∝ higher volume/BSA, MD, *P* = 0.037	^66^
Composite score^a^	• HCC	Higher risk ∝ higher score, HR: 5.99, *P* < 0.01	^28^
Stiffness (ARFI)	• Death, heart transplantation, ventricular assist device implantation, decompensated heart failure	Higher risk ∝ higher stiffness, MD, *P* = 0.04	^67^
	• Thromboembolic events	Higher risk ∝ higher stiffness, OR: 2.12, *P* = 0.03	^68^
	• Death, heart transplantation, HCC, varices	Not a predictor, MD, *P* = 0.127	^11^
MRI			
Liver volume	• Death, heart transplantation, ventricular assist device implantation, nonelective cardiovascular hospitalization • Death, heart transplantation, decompensated heart failure	Higher risk ∝ higher volume, MD, *P* = 0.01 Not a predictor, MD, *P* = 0.28	^69^ ^24^
Stiffness	• Death, heart transplantation, decompensated heart failure	Higher risk ∝ higher stiffness, MD, *P* = 0.03	^24^
Cirrhosis^b^	• Death, listing for heart transplantation, arrhythmias, protein-losing enteropathy, decompensated heart failure	Not a predictor, chi-square, *P* = 0.77	^49^
Biopsy			
CHFS grade 3-4	• Death	Higher risk ∝ higher biopsy grade, MD, *P* = 0.027	^42^
Combined portal sinusoidal fibrosis	• Protein-losing enteropathy	Higher risk ∝ more fibrosis, chi-square, *P* = 0.003	^70^
Centrilobular fibrosis Portal fibrosis	• Death, heart transplantation • Death, heart transplantation	Not a predictor, OR: 1.3, *P* = 0.69 Not a predictor, OR: 0.6, *P* = 0.38	^25^ ^25^
Mixed			
Cirrhosis^c^	• Death	Higher risk ∝ cirrhosis, HR: 2.7, *P* = 0.08	^71^
Cirrhosis^d^	• HCC	Higher risk ∝ cirrhosis, chi-square, *P* < 0.01	^72^
Cirrhosis^e^	• Arrhythmias • Thromboembolic events	Higher risk ∝ cirrhosis, OR: 6.9, *P* = 0.06 Higher risk ∝ cirrhosis, OR: 5.4, *P* = 0.04	^46^ ^46^
Cirrhosis^f^	• Protein-losing enteropathy	Not a predictor, chi-square, *P* = 0.12	^16^
Composite score^g^	• Death	Higher risk ∝ higher score, chi-square, *P* = 0.018	^41^
VAST ≥2^h^	• Death, heart transplantation, HCC • Death, heart transplantation, ventricular assist device implantation, decompensated heart failure	Higher risk ∝ VAST ≥2, OR: 9.8, *P* < 0.05 Higher risk ∝ VAST ≥2, OR: 10.2, *P* = 0.003	^62^ ^33^

ARFI = acoustic radiation force impulse; BSA = body surface area; AST = aspartate aminotransferase; CHFS = congestive hepatic fibrosis score; GGT = gamma glutamyl transferase; HCC = hepatocellular carcinoma; MD = mean difference in liver imaging marker between those who reached the endpoint or not; MRI = magnetic resonance imaging; VAST = Varices, Ascites, Splenomegaly or Thrombocytopenia.

a Parenchymal echotexture (normal = 0, coarse = 1), surface irregularity (smooth = 0, irregular = 1), ascites (nonsmall = 0, ≥ moderate = 1), number of hyperechoic spots (≥3 mm in diameter; none to a few = 0, larger number = 1), and space-occupying lesions (no = 0, yes = 1).

b Both lobulation and nodularity.

c Liver stiffness >5 kPA on magnetic resonance elastography or stage 4 fibrosis on biopsy.

d Biopsy or imaging features plus varices and splenomegaly.

e Undefined.

f Biopsy or imaging features (parenchymal heterogeneity with irregular undulating liver margins and caudate hypertrophy with or without enhancing nodules).

g Ultrasound (surface nodularity/blunted liver edge, heterogenous parenchyma/echo bright lesions, ascites, splenomegaly, abnormal portal vein flow) and laboratory abnormalities (thrombocytopenia, elevated GGT, elevated AST, prothrombin activity <70%, and hypoalbuminaemia).

h Varices, ascites, splenomegaly, and platelet ≤150.

### Sensitivity analysis and publication bias

Sensitivity analysis using the “leave-one-out” method revealed that no study had a disproportionate impact on the primary outcome (Supplemental Figure 8). There were no publication biases as visually assessed by the funnel plots and confirmed statistically by Begg test (Supplemental Figure 9).

## Conclusions

FALD encompasses a spectrum of liver disease that is commonly seen in patients living with Fontan circulation. Despite increasing recognition, there remain significant practice differences in screening for FALD, likely stemming from inconsistent data on its risk factors, diagnostic method, and prognostic significance. The current study is the first systematic attempt to provide an unbiased summary of prevalence, diagnosis, risk factors, and prognosis of FALD in those routinely screened. Despite this, the key findings of our review reflect the significant heterogeneity in outcomes as a result of variable institutional practices and patient factors (Central Illustration). Importantly, we have noted various factors contributing toward such heterogeneity including diagnostic method, time after Fontan completion, type of Fontan circulation, ventricular dysfunction, and PCWP. These factors should be considered in clinical practice in evaluating the pretest probability of FALD in individual patients, as well as in designing structured screening programs.

**Central Illustration:**
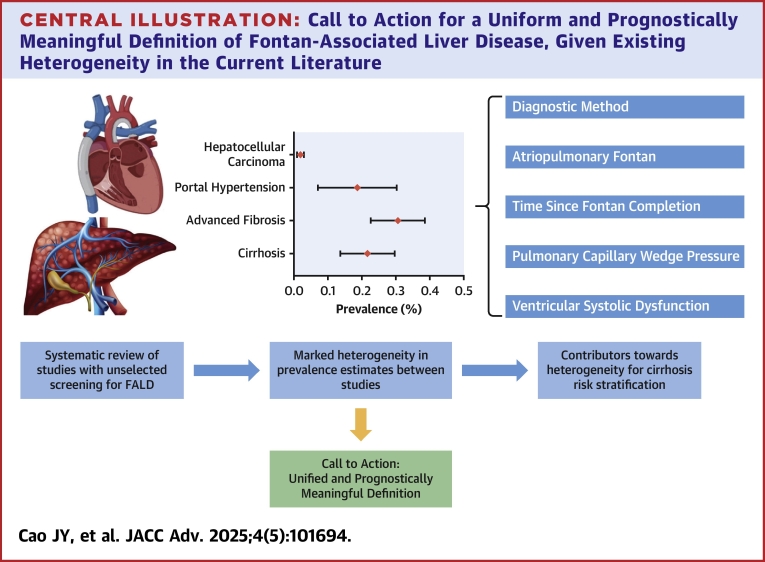
Call to Action for a Uniform and Prognostically Meaningful Definition of Fontan-Associated Liver Disease, Given Existing Heterogeneity in the Current Literature

Prevalence of FALD differed markedly between studies. Indeed, between the 26 studies reporting cirrhosis, we noted 8 different definitions of cirrhosis, which should serve as impetus for more unifying definitions of FALD in future studies. Despite attempting to minimize the confounding effect of patient selection on risk of liver disease by excluding studies with selective liver screening, the reported cirrhosis rate still varied from 0 to 76%, and likewise for advanced fibrosis, portal hypertension, and HCC. Large cohort studies were not immune to such variations, suggesting that the difference is likely underpinned by diagnostic method and criteria rather than random variations. The largest cohort consisted of 1,117 Japanese Fontan patients, and estimated incidence of biopsy or imaging-proven cirrhosis was 4% at 15 years post-Fontan completion.^8^ In contrast, a multicenter Korean study of 313 patients who were routinely screened for liver disease via ultrasound or computed tomography noted just over half of all patients having cirrhosis at 20 years follow-up post-Fontan completion.^15^

Given the lack of consensus on the most practical and prognostically meaningful definition of FALD, a spectrum of invasive and noninvasive tools has been evaluated. Serological tests are often of questionable prognostic significance in FALD and thus excluded from our analyses. Liver function tests are commonly performed but do not correlate well with the severity of liver injury.^16^^,^^17^ More nuanced serological panels that predict cirrhosis in viral hepatitis have not proven to be useful for FALD. The aspartate aminotransferase-to-platelet ratio score and the Fibrosis-4 score were demonstrated in a cohort of 159 adult Fontan patients to be independently predictive of all-cause mortality, although correlated poorly with fibrosis severity on biopsy and features of portal hypertension.^18^ Recent data from the Fontan Outcomes Study to Improve Transplant Experience and Results Registry suggested that no commonly used serological markers differentiated between those receiving an isolated heart vs combined heart and liver transplant, whereas clinical and imaging markers of cirrhosis or portal hypertension did.^19^ MELD-XI has been shown to correlate with the degree of biopsy-proven fibrosis in a case series of 70 stable Fontan patients.^20^ Furthermore, it was predictive in another series of a composite cardiac endpoint of heart failure mortality, sudden death, and cardiac transplantation^21^, although its association with liver-related clinical outcomes was not evaluated. Our meta-regression did not identify a relationship between MELD-XI and liver outcomes, although the analysis was limited by only 8 studies reporting cirrhosis and MELD-XI. Noninvasive elastography techniques also are of questionable value in FALD due to the reliance on liver stiffness, which is elevated in hepatic congestion independently of the presence of fibrosis.^22^ Given the variability in the elastography techniques used (transient elastography, magnetic resonance elastography, and acoustic resonance force impulse imaging) as well as the cutoff values used for cirrhosis in the included studies, we were unable to quantitatively summarize the utility of elastography in FALD.

Our study demonstrated a more than 2-fold higher diagnosis rate of cirrhosis by imaging than by biopsy. Hepatic imaging has traditionally been performed via ultrasound or computed tomography. However, liver parenchymal abnormalities, such as nodularity, are common and do not necessarily reflect histological advanced fibrosis or cirrhosis.^2^ Magnetic resonance imaging has recently emerged as a more sensitive and reproducible imaging modality and has been shown to correlate with portal hypertension, liver biopsy, and clinical outcomes.^23^^,^^24^ Biopsy remains the gold standard but is limited by procedural risks and the patchy nature of fibrosis in FALD, which can be missed in insufficient sampling.^25^ Various biopsy scoring tools used in the included studies have focused on either periportal or sinusoidal fibrosis. It is likely that both processes occur in FALD and should be considered together in assessing liver disease.^26^^,^^27^

Various FALD risk factors have been inconsistently highlighted in existing literature. The current study-level meta-regression offers a more holistic overview, identifying time post-Fontan completion, AP Fontan, PCWP, and ventricular dysfunction to be significant predictors of cirrhosis. The lack of a subpulmonic ventricle post-Fontan completion exposes the liver to chronically elevated CVP, and over time, this results in congestive hepatopathy and cirrhosis. This is reflected in the latency for cirrhosis to manifest, with most patients diagnosed more than 10 years post-Fontan completion as noted in our study and previous large cohorts.^8^^,^^15^ This process may be accelerated by presence of ventricular dysfunction, as evidenced through elevated PCWP, also highlighted in our analyses. Consequently, there is increasing interest in modifiable risk factors and surrogate markers for early identification, risk stratification, and management. Importantly, right heart catheterization was done at different times between the included studies, with some around the time of cirrhosis diagnosis, which would diminish their value as predictors. In a cohort of over 1,000 Fontan patients who underwent routine catheterization at a median of 1 year post-Fontan, it was found that every 3 mm Hg increase in CVP was associated with a 31% increased hazard of developing cirrhosis and HCC at long-term follow-up.^8^ The current results also support the shift away from atriopulmonary Fontan, which has been demonstrated to have worse overall survival and increased risk of arrhythmias.^1^

The presence of FALD is prognostic of liver and cardiac outcomes. As summarized in Table 3, several studies have described associations between FALD and overall survival. In the Fontan Outcomes Study to Improve Transplant Experience and Results Registry data, the authors found that isolated heart transplantation was noninferior to combined heart-liver transplantation in those with minimal clinical features of cirrhosis or portal hypertension at listing assessment. In contrast, in those with at least 2 features (imaging-proven cirrhosis, varices, splenomegaly, and recurrent ascites), combined heart-liver transplant was associated with better survival, suggesting the pretransplant liver disease to be a predictor of outcomes.^19^ As highlighted in our meta-analysis, HCC is a rare complication even at long-term follow-up. Once diagnosed, however, it does carry a dismal outcome with a 1-year survival of approximately 50%.^28^^,^^29^, ^30^, ^31^ Interestingly, in North American, European, and Japanese cohorts, only half of patients with HCC had a preceding diagnosis of cirrhosis, much lower than non-Fontan patients with HCC.^32^ Whether this represents different pathological processes contributing to carcinogenesis or an underdiagnosis of cirrhosis is unclear.

With regard to management strategies, there is theoretical benefit in decreasing ventricular filling pressure, reducing pulmonary vascular resistance, or identifying and relieving anatomical obstructions. However, there have been limited studies in these domains with inconsistent outcomes.^12^ This may relate to small cohort sizes with short follow-up or selection of patients with irreversible liver disease, but importantly underscores the need for large prospective studies to better understand the mechanism of FALD in order to design more targeted therapies.

### Study limitations

The results of the current study should be interpreted with caveats. Firstly, there is significant heterogeneity in outcomes. The current study, however, is not an attempt to offer a single definitive prevalence estimate. Rather, it recognizes and explores the underlying reason for such heterogeneity, which also exists in clinical practice, and offers potential reasons (eg, diagnostic method and risk factor profiles) that may help to stratify patients for FALD screening. Nevertheless, a more homogenous dataset would possess a higher internal validity, although this was not available for the current review. Secondly, many of the studies did not define HCC diagnosis or diagnosed HCC by imaging, which is not validated in a Fontan cohort. This may have overestimated the prevalence compared to if the diagnosis was histological only. Thirdly, data regarding risk factors for other forms of liver disease were rarely reported, and hence we were unable to control for the presence of coexisting primary liver disorders or lifestyle-related risk factors such as steatotic liver diseases. Finally, we did not quantitatively evaluate the prognostic significance of FALD on the basis of variations in analyzed variables and analysis methods between studies.

## Conclusions

FALD is a highly heterogeneous syndrome that is common after Fontan completion. We have attempted to perform a comprehensive and unbiased meta-analysis of the prevalence and potential risk factors for FALD. More importantly, we have highlighted an inherent issue in the FALD literature, which is the need for large prospective studies with prognostic, validated, and homogenous definitions of liver disease in Fontan patients in order to design better screening and preventative protocols.

Perspectives **COMPETENCY IN MEDICAL KNOWLEDGE 1:** FALD should be considered in all patients after 5 to 10 years post-Fontan completion. There is currently no single validated diagnostic test, so a multimodal approach, interpreted in the clinical context, should be adopted.

**COMPETENCY IN MEDICAL KNOWLEDGE 2:** FALD is a prognostic marker of poor overall outcome and therefore should be considered in risk stratification of this unique group of patients.

**TRANSLATIONAL OUTLOOK 1:** Diagnostic methods commonly applied to other causes of chronic liver disease may not be valid in FALD. As such, future research should aim to evaluate these and identify novel and prognostically meaningful markers of disease.

## Funding support and author disclosures

The authors have reported that they have no relationships relevant to the contents of this paper to disclose.
