# Supplementary material for: Prevalence, Risk Factors, and Prognosis for Fontan-Associated Liver Disease: A Systematic Review and Exploratory Meta-Analysis
Source: JACC Adv. 2025 Apr 25;4(5):101694. doi: 10.1016/j.jacadv.2025.101694 (PMC12101536; doi:10.1016/j.jacadv.2025.101694)
Supplement: Supplemental_Material [file mmc1.docx]

**Supplemental Appendix**

Search Strategy

| 1 | (fontan or single ventric* or univentric* or total cavopulmonary anastomosis or lateral tunnel or atriopulmonary).tw. |
| --- | --- |
| 2 | exp Fontan Procedure/ |
| 3 | cirrho*.tw. |
| 4 | (liver adj6 fibro*).tw. |
| 5 | (hepatic adj6 fibro*).tw. |
| 6 | portal hypertension.tw. |
| 7 | hcc.tw. |
| 8 | (liver adj6 cancer*).tw. |
| 9 | hepatocellular carcinoma*.tw. |
| 10 | (liver adj6 disease*).tw. |
| 11 | exp Liver Cirrhosis/ |
| 12 | exp Hypertension, Portal/ |
| 13 | exp Carcinoma, Hepatocellular/ |
| 14 | 3 or 4 or 5 or 6 or 7 or 8 or 9 or 10 or 11 or 12 or 13 |
| 15 | 1 or 2 |
| 16 | 14 and 15 |


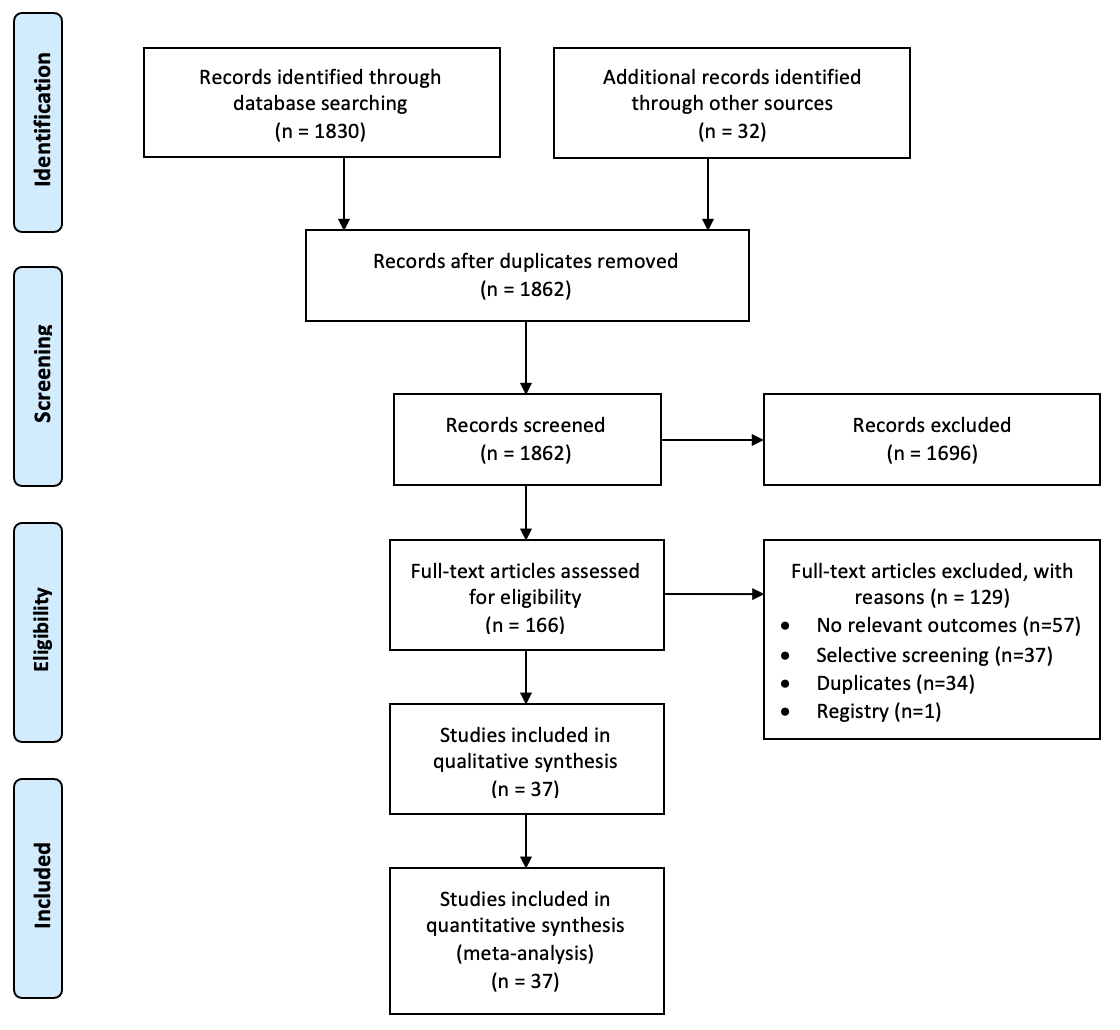


**Supplemental Figure 1.** Preferred Reporting Items for Systematic Reviews and Meta-Analyses flow chart for search results


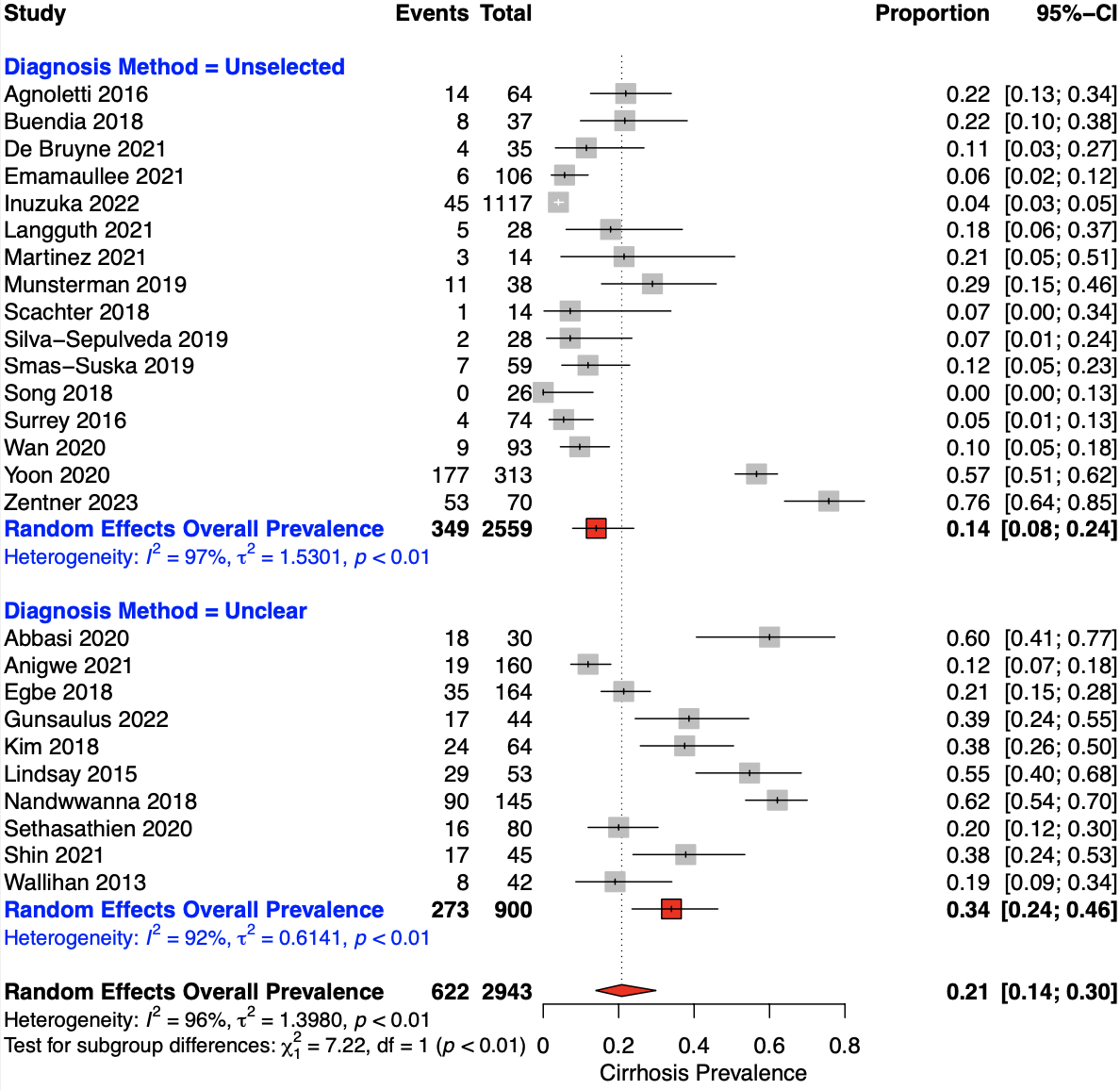


**Supplemental Figure 2.** Prevalence of cirrhosis grouped according to patient selection method – unselected recruitment versus undefined recruitment methods. A higher prevalence of cirrhosis was noted in studies with undefined recruitment methods.

**
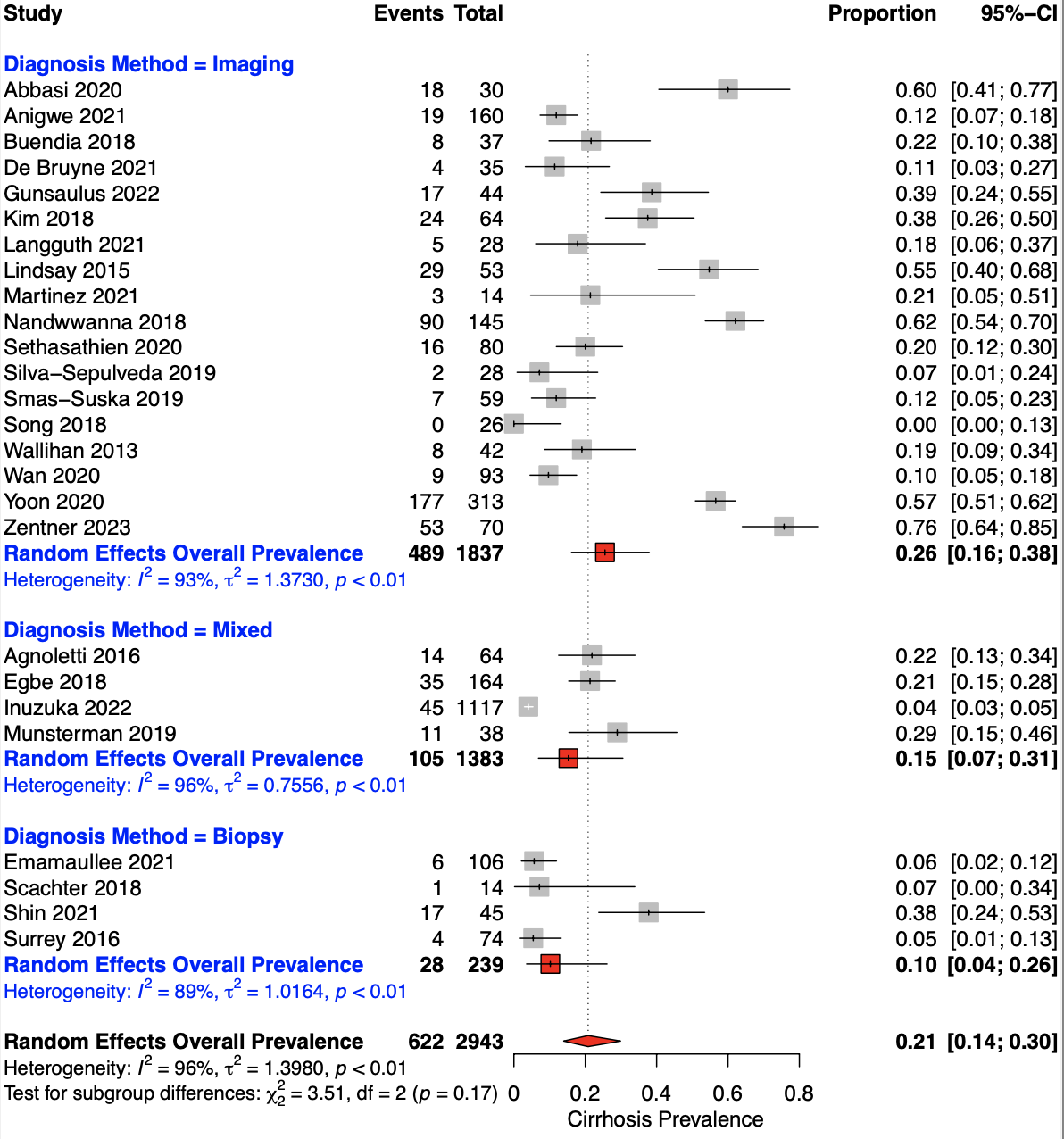
**

**Supplemental Figure 3.** Prevalence of cirrhosis grouped according to diagnostic method – biopsy, imaging or mixture of both. A lower prevalence of cirrhosis was noted in studies using only biopsy than using only imaging, with mixed studies reporting prevalence in between.

** Supplemental Figure 4.** Positive correlation between PCWP and cirrhosis prevalence. PCWP = pulmonary capillary wedge pressure.

** Supplemental Figure 5.** Positive correlation between proportion of atriopulmonary and cirrhosis prevalence.

** Supplemental Figure 6.** Positive correlation between proportion of moderate or greater ventricular dysfunction and cirrhosis prevalence.

** Supplemental Figure 7.** Trend towards positive correlation between proportion of central venous pressure and cirrhosis prevalence.

**Supplemental Figure 8.** Sensitivity analysis using the leave-one-out method for the primary outcome of cirrhosis.


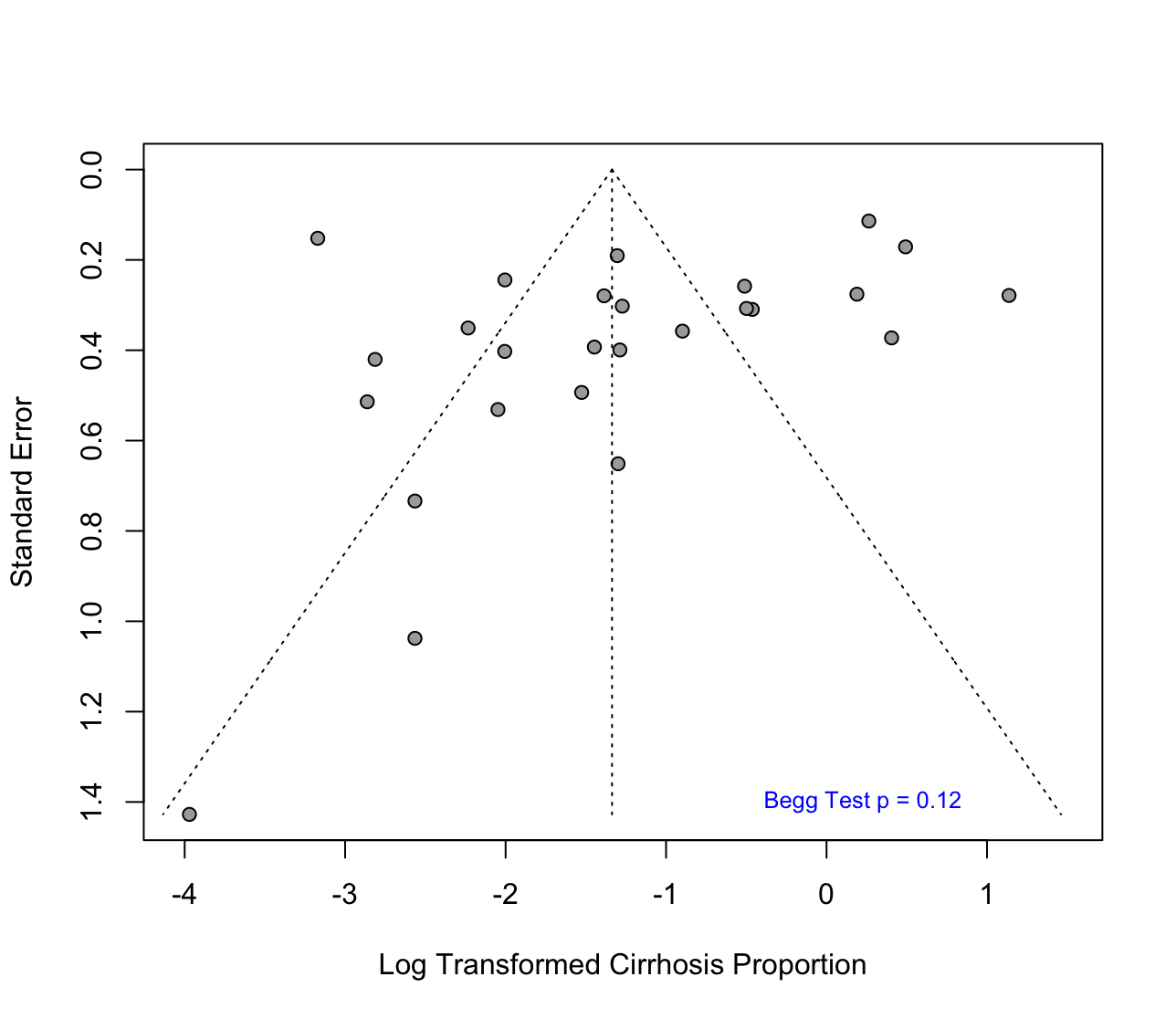

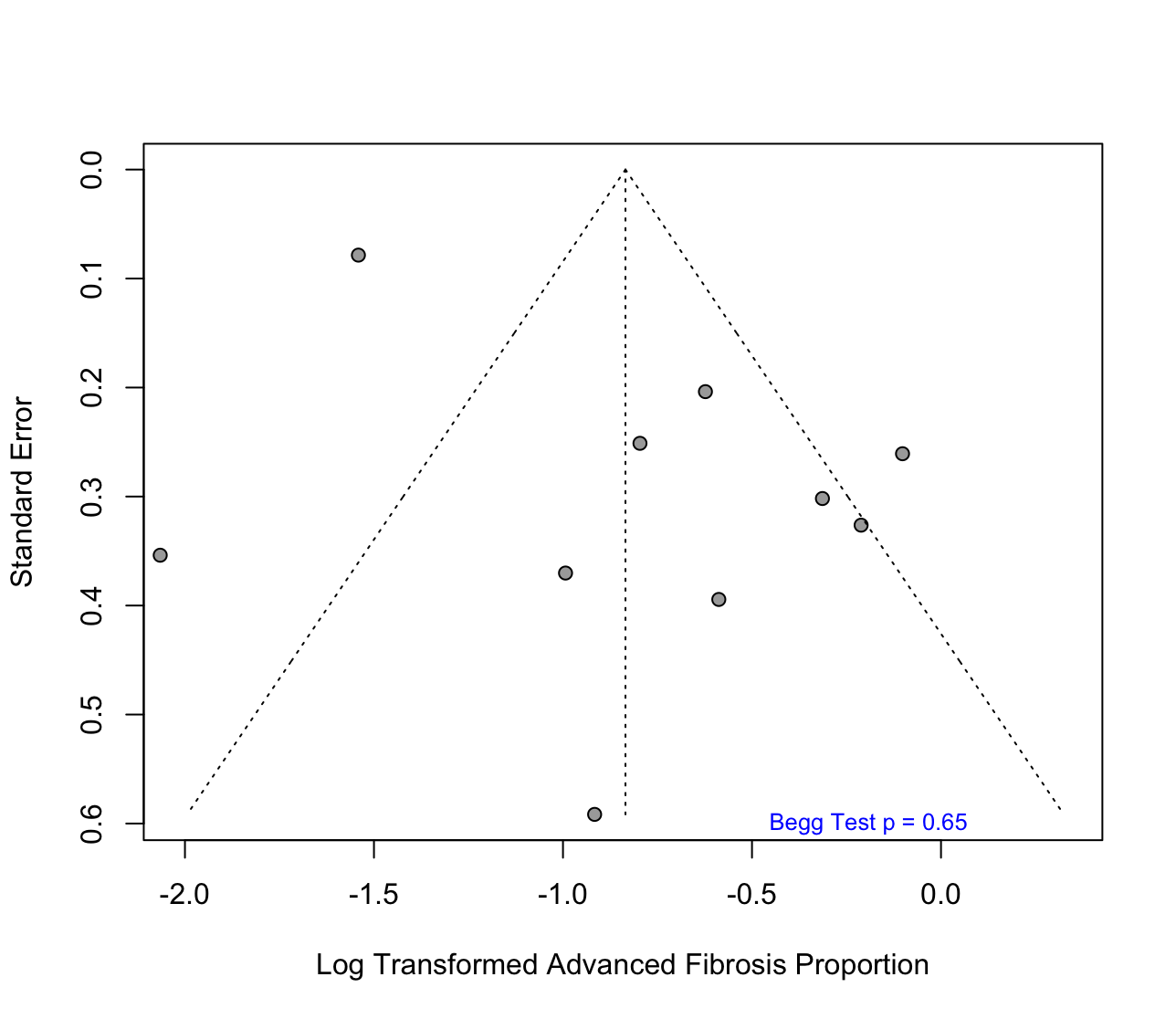

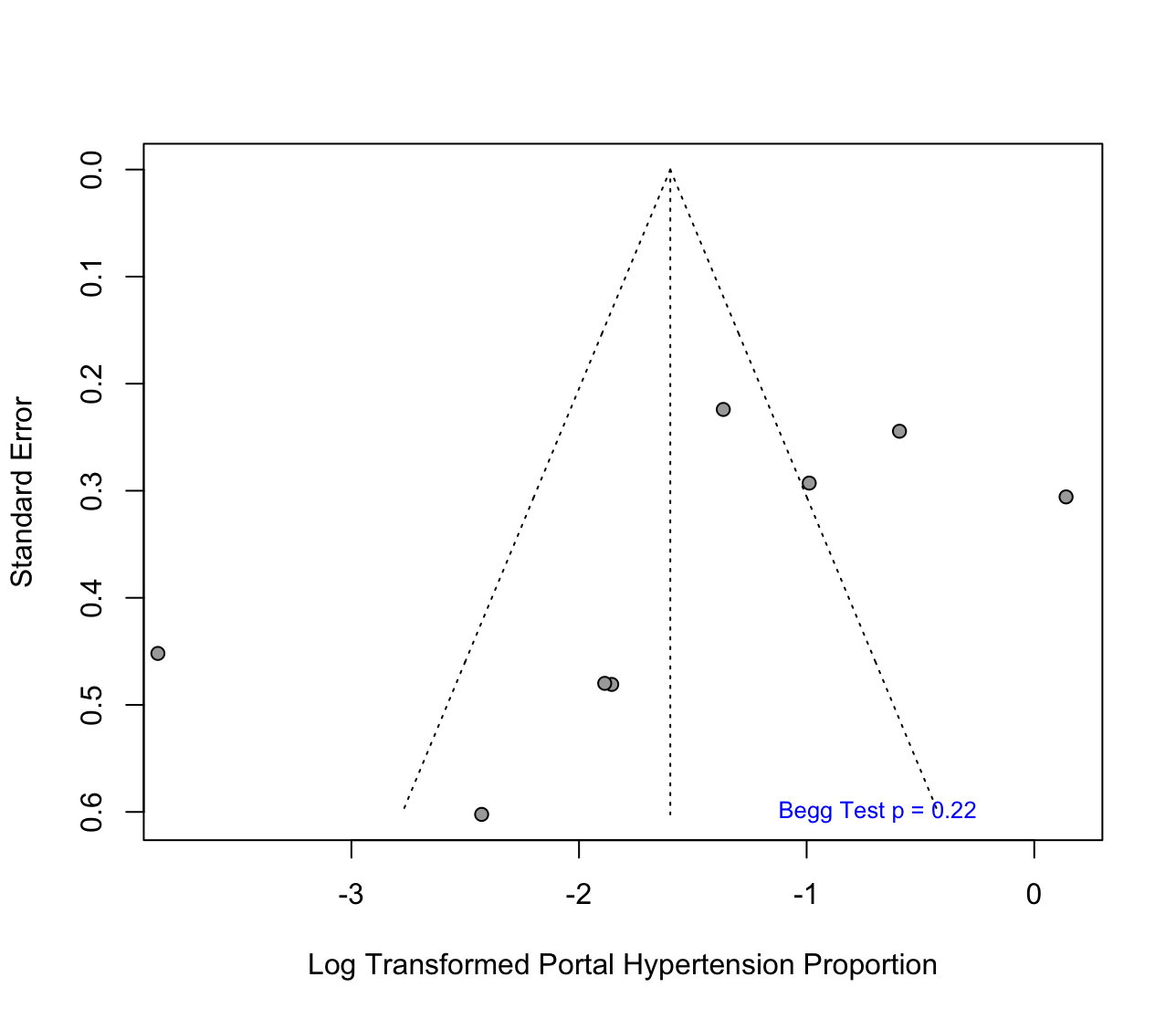

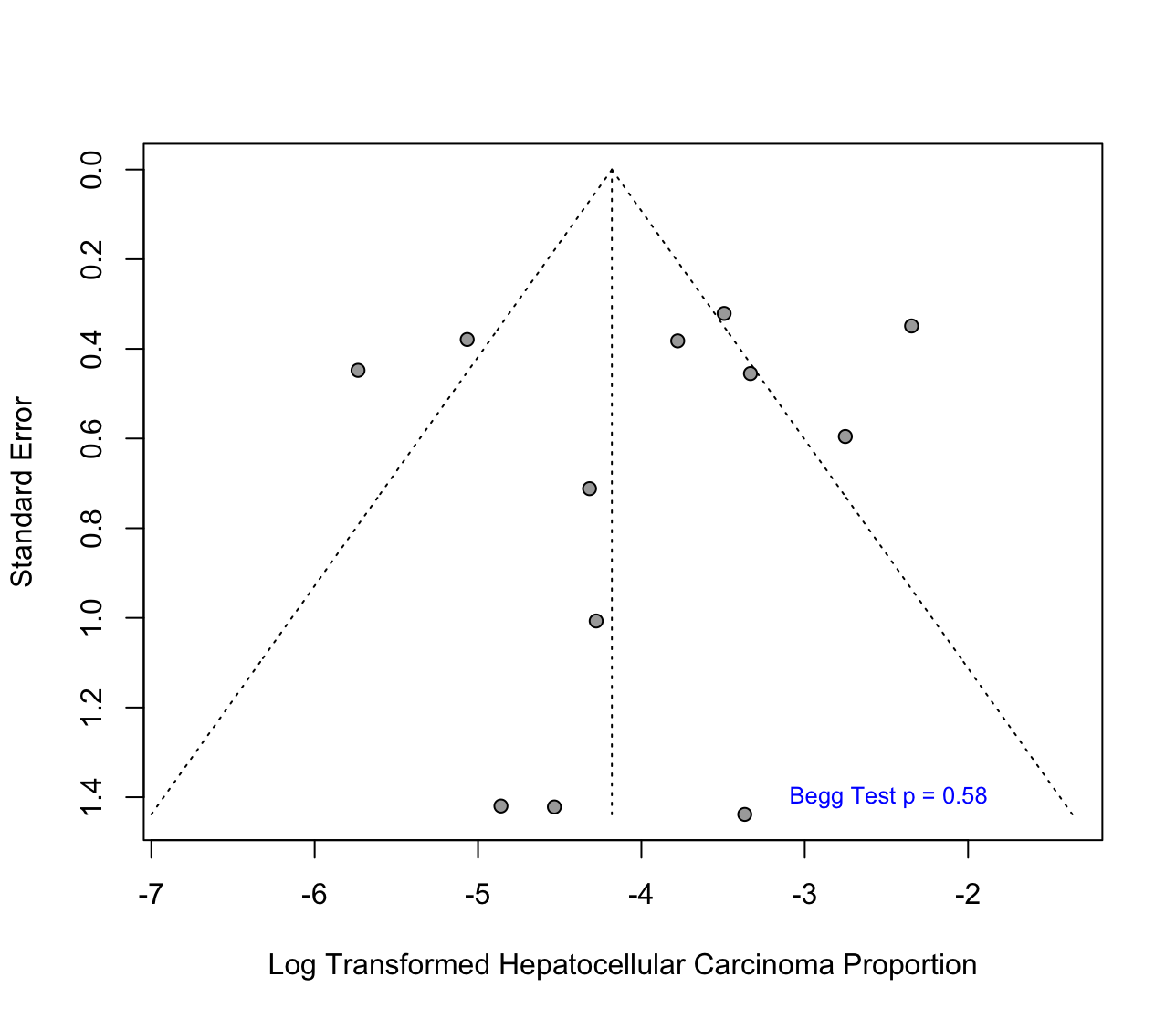


**Supplemental Figure 9.** Funnel plots and associated Begg’s test to evaluate risk of publication bias in the four outcomes.

| **Study** | **Cirrhosis Definition** | **Advanced Fibrosis Definition** | **Portal Hypertension Definition** |
| --- | --- | --- | --- |
| Zentner 2023 | Imaging: ARFI > 1.8 m/s |  |  |
| Zafar 2022 |  |  | ≥ 2 of varices, ascites, splenomegaly |
| Inuzuka 2022 | Biopsy: Scheuer Stage 4 or METAVIR Stage 4  Imaging: US/CT (presence of contour nodularity, blunt margins, right hepatic lobe atrophy, caudate or left hepatic lobe hypertrophy) or portal hypertension features (varices, splenomegaly) | Biopsy: Scheuer Stage 2-3 or METAVIR Stage 2-3  Imaging: US (blunted liver edge, densely dotted high echogenicity or coarse parenchymal echotexture) or CT (blunted liver edge or parenchymal enhancement with reticular or mosaic perfusion in portal venous phase without features of cirrhosis) |  |
| Gunsaulus 2022 | Imaging: nodular liver surface and hypoechoic nodules in the liver parenchyma |  |  |
| Shin 2021 | Biopsy: Ludwig F4 | Biopsy: Ludwig F3 |  |
| Navallas 2021 |  |  | Either portosystemic varices alone or splenomegaly plus ascites |
| Martinez 2021 | Imaging: heterogeneous echogenicity or nodular surface |  |  |
| Langguth 2021 | Imaging: 3 features of liver edge bluntness, liver surface nodularity, ascites, splenomegaly | Imaging: 2 features of liver edge bluntness, liver surface nodularity, ascites, splenomegaly |  |
| Hansen 2021 | Mixed: combined liver US (surface nodularity, heterogenous parenchyma, abnormal portal vein flow, splenomegaly, ascites) and serology score (thrombocytopaenia, elevated gamma-glutamyl transpeptidase, elevated aminotransferase, reduced prothrombin time, hypoalbuminaemia) ≥ 5 | Imaging: combined liver US (surface nodularity, heterogenous parenchyma, abnormal portal vein flow, splenomegaly, ascites) and serology score (thrombocytopaenia, elevated gamma-glutamyl transpeptidase, elevated aminotransferase, reduced prothrombin time, hypoalbuminaemia) total 3-4 | VAST ≥ 2 |
| Emamaullee 2021 | Biopsy: Congestive Hepatic Fibrosis Score Stage 4 | Biopsy: Congestive Hepatic Fibrosis Score Stage 3 |  |
| De Bruyne 2021 | Imaging: irregularity of liver surface, or nodularity of liver parenchyma |  |  |
| Chemello 2021 |  |  | At least 2 of the main criteria indicative of advanced liver disease, among following: spleen diameter >120 mm, portal vein diameter >12 mm, portal flow rate <12 cm/sec or presence of gastro-oesophageal varices at esophagogastroduodenoscopy or of collateral circuits at imaging techniques |
| Anigwe 2021 |  |  |  |
| Yoon 2020 | Imaging: concurrent presence of liver surface nodularity and signs of portal hypertension, including presence of splenomegaly (defined as size greater than 12 cm in the largest bipolar diameter at the splenic hilum), presence of ascites, and/or presence of portosystemic collateral vessels (i.e., varices) |  |  |
| Wan 2020 |  |  |  |
| Sethasathien 2020 |  | Imaging: elastography > 15 kPa |  |
| Abbasi 2020 | Imaging: lobulation and nodularity present |  |  |
| Smas-Suska 2019 | Imaging: SWE diagnostic stages (unspecified thresholds) |  | VAST ≥ 2 |
| Silva-Sepulveda 2019 |  |  |  |
| Munsterman 2019 | Biopsy: Congestive Hepatic Fibrosis Score Stage 4 | Biopsy: Congestive Hepatic Fibrosis Score Stage 3 | Presence of all three of ascites, varices and splenomegaly |
| Song 2018 | Imaging: contour nodularity, blunt margins, right hepatic lobe atrophy, caudate or left hepatic lobe hypertrophy, and extrahepatic manifestations of portal hypertension (enlarged spleen and enlarged portal vein). |  |  |
| Scachter 2018 | Biopsy: Congestive Hepatic Fibrosis Score Stage 4 | Biopsy: Congestive Hepatic Fibrosis Score Stage 3 |  |
| Nandwwanna 2018 | Imaging: coarsened or heterogenous echotexture (US), lobar redistribution (CT/MRI), evidence of fibrosis (CT/MRI), or evidence of portal hypertension such as splenomegaly or ascites due to chronic liver disease (US, CT/MRI), and varices (CT/MRI) without HCC |  |  |
| Kim 2018 | Imaging: ARFI > 2 m/s |  |  |
| Egbe 2018 | Biopsy: stage 4 fibrosis on histology Imaging: liver stiffness >5.0 kPa by magnetic resonance elastography |  |  |
| Buendia 2018 | Imaging: blunt margins, right hepatic lobe atrophy, contour nodularity, caudate or left hepatic lobe hypertrophy, and extrahepatic manifestations of portal hypertension (ascites, collateral circulation, and splenomegaly) |  | Splenomegaly or ascites |
| Surrey 2016 | Biopsy: Congestive Hepatic Fibrosis Score Stage 4 or METAVIR Stage 4 | Biopsy: Congestive Hepatic Fibrosis Score Stage 3 or METAVIR Stage 3 |  |
| Agnoletti 2016 | Biopsy: liver cirrhosis  Imaging: sonographic evidence of cirrhosis with platelet count ≤ 100,000/mm^3^  Mixed: evidence of cirrhosis-related complications, such as ascites, varices or hepatic encephalopathy |  |  |
| Lindsay 2015 | Imaging: computed tomography or magnetic resonance imaging findings of parenchymal heterogeneity with irregular undulating liver margins, and caudate hypertrophy with or without enhancing nodules |  |  |
| Wallihan 2013 | Imaging: findings of cirrhosis including contour nodularity, blunt margins, right hepatic lobe atrophy, caudate or left hepatic lobe hypertrophy, and extrahepatic manifestations of portal hypertension |  |  |
| Elder 2013 |  |  | VAST ≥ 2 |

**Supplemental Table 1.** Definition of cirrhosis, advanced fibrosis and portal hypertension. ARFI = acoustic radiation force impulse; CT = computed tomography; HCC = hepatocellular carcinoma; MRI = magnetic resonance imaging; SWE = shear wave elastography; US = ultrasound; VAST = Varices, Ascites, Splenomegaly, or Thrombocytopenia

| **Diagnostic Method** | **Endpoint** | **Association, Effect Estimator/Size, P value** |  |
| --- | --- | --- | --- |
| **Imaging** |  |  |  |
| Ultrasound |  |  |  |
| Liver volume/BSA | - Death, heart/liver transplantation | Higher risk ∝ higher volume/BSA, MD, *p* = 0.037 | ^32^ |
| Composite score^1^ | - HCC | Higher risk ∝ higher score, HR 5.99, *p* < 0.01 | ^30^ |
| Stiffness (ARFI) | - Death, heart transplantation, ventricular assist device implantation, decompensated heart failure | Higher risk ∝ higher stiffness, MD, *p* = 0.04 | ^33^ |
|  | - Thromboembolic events | Higher risk ∝ higher stiffness, OR 2.12, *p* = 0.03 | ^34^ |
|  | - Death, heart transplantation, HCC, varices | Not a predictor, MD, *p* = 0.127 | ^10^ |
| MRI |  |  |  |
| Liver volume | - Death, heart transplantation, ventricular assist device implantation, nonelective cardiovascular hospitalisation - Death, heart transplantation, decompensated heart failure | Higher risk ∝ higher volume, MD, *p* = 0.01  Not a predictor, MD, *p* = 0.28 | ^35^  ^22^ |
| Stiffness | - Death, heart transplantation, decompensated heart failure | Higher risk ∝ higher stiffness, MD, *p* = 0.03 | ^22^ |
| Cirrhosis^2^ | - Death, listing for heart transplantation, arrhythmias, protein losing enteropathy, decompensated heart failure | Not a predictor, χ^2^, *p* = 0.77 | ^36^ |
| **Biopsy** |  |  |  |
| CHFS grade 3-4 | - Death | Higher risk ∝ higher biopsy grade, MD, *p* = 0.027 | ^37^ |
| Combined portal sinusoidal fibrosis | - Protein losing enteropathy | Higher risk ∝ more fibrosis, χ^2^, *p* = 0.003 | ^38^ |
| Centrilobular fibrosis  Portal fibrosis | - Death, heart transplantation - Death, heart transplantation | Not a predictor, OR = 1.3, *p* = 0.69  Not a predictor, OR = 0.6, *p* = 0.38 | ^39^  ^39^ |
| **Mixed** |  |  |  |
| Cirrhosis^3^ | - Death | Higher risk ∝ cirrhosis, HR 2.7, *p* = 0.08 | ^40^ |
| Cirrhosis^4^ | - HCC | Higher risk ∝ cirrhosis, χ^2^, *p* < 0.01 | ^41^ |
| Cirrhosis^5^ | - Arrhythmias - Thromboembolic events | Higher risk ∝ cirrhosis, OR 6.9, *p* = 0.06  Higher risk ∝ cirrhosis, OR 5.4, *p* = 0.04 | ^42^  ^42^ |
| Cirrhosis^6^ | - Protein losing enteropathy | Not a predictor, χ^2^, *p* = 0.12 | ^16^ |
| Composite score^7^ | - Death | Higher risk ∝ higher score, χ^2^, *p* = 0.018 | ^43^ |
| VAST^8^ ≥ 2 | - Death, heart transplantation, HCC - Death, heart transplantation, ventricular assist device implantation, decompensated heart failure | Higher risk ∝ VAST ≥ 2, OR 9.8, *p* < 0.05  Higher risk ∝ VAST ≥ 2, OR 10.2, *p* = 0.003 | ^44^  ^45^ |

**Supplemental Table 2.** Summary of studies evaluating prognostic role of liver disease in Fontan patients. ARFI = acoustic radiation force impulse. BSA = body surface area. CHFS = congestive hepatic fibrosis score. HCC = hepatocellular carcinoma. HR = hazard ratio. MD = mean difference in liver imaging marker between those who reached the endpoint or not. MRI = magnetic resonance imaging. OR = odds ratio. ^1^ Parenchymal echotexture (normal=0, coarse=1), surface irregularity (smooth=0, irregular=1), ascites (non-small=0, ≥ moderate=1), number of hyperechoic spots (≥3 mm in diameter; none to a few=0, larger number=1), and space-occupying lesions (no=0, yes=1). ^2^ Both lobulation and nodularity. ^3^ Liver stiffness >5kPA on magnetic resonance elastography; or stage 4 fibrosis on biopsy. ^4^ Biopsy or imaging features plus varices and splenomegaly. ^5^ Undefined. ^6^ Biopsy or imaging features (parenchymal heterogeneity with irregular undulating liver margins, and caudate hypertrophy with or without enhancing nodules). ^7^ Ultrasound (surface nodularity/blunted liver edge, heterogenous parenchyma/echo bright lesions, ascites, splenomegaly, abnormal portal vein flow) and laboratory abnormalities (thrombocytopaenia, elevated GGT, elevated AST, prothrombin activity < 70%, hypoalbuminaemia). ^8^ Varices, ascites, splenomegaly, and platelet ≤ 150
